# Common mechanisms of Wumei pills in treating ulcerative colitis and type 2 diabetes: Exploring an integrative approach through network pharmacology

**DOI:** 10.1097/MD.0000000000037094

**Published:** 2024-01-26

**Authors:** Chang Sun, Keyuan Xiao, Yinxiong He, Xinghua Li

**Affiliations:** a Department of United Front Work, Heilongjiang University of Chinese Medicine, Harbin, Heilongjiang, China; b Changzhi People’s Hospital Affiliated to Changzhi Medical College, Changzhi, China; c Graduate school, Heilongjiang University of Chinese Medicine, Harbin, Heilongjiang, China.

**Keywords:** integrative treatment, network pharmacology, type 2 diabetes, ulcerative colitis, Wumei pills

## Abstract

Wumei pills (WMP), a classical Chinese herbal formula, have shown efficacy in the treatment of ulcerative colitis (UC) and type 2 diabetes (T2DM). However, the underlying mechanisms by which WMP simultaneously targets these distinct diseases remain unclear. In this study, a network pharmacology approach was employed to unravel the potential molecular mechanisms of WMP in UC and T2DM treatment. This analysis provides a bioinformatics foundation for the traditional Chinese medicine concept of “treating different diseases with the same treatment.” WMP was found to contain 65 active components, including flavonoids, sterols, and alkaloids, that act on 228 shared targets for UC and T2DM. Network analysis identified 5 core compounds (Quercetin, Kaempferol, beta-Sitosterol, Isocorypalmine, Stigmasterol) and 8 core proteins (AKT1, ESR1, TP53, IL6, JUN, MYC, TNF, EGFR) that play pivotal roles in the treatment of UC and T2DM by WMP. WMP exerts its therapeutic effects by modulating signaling pathways, including the NF-κB pathway, PI3K-Akt pathway, and p53 pathway. Molecular docking results indicate a strong binding affinity between core compounds and core genes. This study bridges the understanding of 2 diseases using network pharmacology and provides insights into shared therapeutic mechanisms, opening doors for further research in modern Chinese herbal formulations.

## 1. Introduction

Ulcerative colitis (UC) is a chronic, nonspecific inflammatory condition that impacts the rectum and colon, its etiology remaining elusive. The condition is marked by gastrointestinal symptoms, including abdominal pain, diarrhea, mucopurulent bloody stools, and tenesmus.^[1]^ Furthermore, UC can result in complications in various organ systems, such as the musculoskeletal system, skin, liver, biliary tract, and eyes.^[2]^ Conversely, type 2 diabetes (T2DM) is a chronic metabolic disorder marked by elevated blood sugar levels resulting from inadequate insulin production or cellular insulin resistance. Clinical manifestations include polydipsia, polyphagia, polyuria, emaciation, and fatigue due to a combination of metabolic dysregulations.^[3]^ T2DM represents more than 90% of all diabetes cases.^[4]^ UC is frequently diagnosed in Chinese young adults, peaking between the ages of 20 and 49.^[5]^ Simultaneously, diabetes is on the rise among younger individuals, showing a prevalence rate of 11.2% in those aged 18 and above from 2015 to 2017. Currently, medications employed for UC and T2DM treatment can alleviate symptoms and decelerate disease progression but frequently carry notable side effects.^[6,7]^

Abundant epidemiological evidence has revealed potential interactions between T2DM and UC.^[8]^ The intestinal tract plays a crucial role in regulating glucose homeostasis, and chronic intestinal inflammation is recognized as a potential risk factor for blood glucose fluctuations in T2DM patients.^[9,10]^ Moreover, T2DM is linked to local and/or systemic chronic inflammation, changes in the gut microbiota, and immune dysfunction.^[11]^ Dysregulation of the intestinal microbiota, impairment of epithelial barrier function, and inflammation may interconnect these 2 diseases.^[12]^ Furthermore, the gut microbiota and its metabolites are implicated in the progression of both T2DM and UC. Alterations in the composition of the gut microbiota structure are closely associated with variations in microbiota-derived metabolites, a connection that has been demonstrated to be intricately involved in the pathogenesis of these 2 diseases.

**Figure 1.**
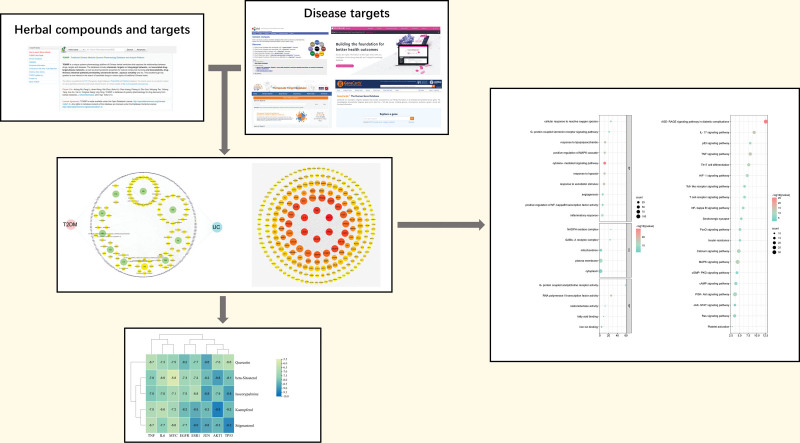
Network pharmacology research flowchart for the treatment of UC and T2DM with WMP. T2DM = type 2 diabetes, UC = ulcerative colitis, WMP = Wumei pills.

Traditional Chinese medicine (TCM) attributes disruptions in the spleen function of transforming and transporting bodily fluids to both UC and T2DM, resulting in the generation of damp-heat. TCM adheres to the principle of “treating different diseases with the same treatment,” applying a uniform therapeutic approach to various diseases due to the emergence of similar pathologies during disease development. The Wumei pills (WMP) under investigation in this study are derived from the “Shanghan Lun”^[13]^ and include 10 medicinal ingredients: *Prunus mume* Siebold & Zucc. (Wumei, WM), *Zingiber officinale* Roscoe (Ganjiang, GJ), *Coptis chinensis* Franch. (Huanglian, HL), *Phellodendron amurense* Rupr. (Huangbai, HB), *Asarum heterotropoides* F. Schmidt (Xixin, XX), *Aconiti Lateralis Radix* Praeparata (Fuzi, FZ), *Zanthoxylum bungeanum* Maxim. (Huajiao, HJ), *Angelica sinensis* (Oliv.) Diels (Danggui, DG), *Cinnamomum cassia* (L.) D. Don (Guizhi, GZ), and *Panax ginseng* C. A. Mey. (Renshen, RS). WMP is recognized for its ability to harmonize the middle, soothe the liver, and clear heat and dampness. Clinical evidence supports the efficacy of WMP in treating both UC and T2DM. Studies have demonstrated that WMP alleviates UC symptoms by impacting inflammatory factors and the gut microbiota.^[14]^ Furthermore, combined treatment with WMP and metformin for T2DM has resulted in significantly better outcomes compared to treatment with metformin alone.^[15]^ These studies substantiate the concept of “treating different diseases with the same treatment.” However, there is limited research on the common mechanisms of WMP in treating both UC and T2DM. This study employs network pharmacology analysis to explore shared action mechanisms, laying a foundation for the investigation of “treating different diseases with the same treatment” in TCM (Fig. 1).

## 2. Methods and materials

### 2.1. Databases and software

The following databases and software tools were used in this study:

AutoDock, CTD (http://ctdbase.org/), Cytoscape 3.9.0, DAVID (https://david.ncifcrf.gov/summary.jsp), DrugBank (https://www.drugbank.ca/), GeneCards (https://genecards.weizmann.ac.il/v3/), Metascape (https://metascape.org/gp/index.html#/main/step1), OpenBabel, PDB (Protein Data Bank, https://www.rcsb.org/), PubChem (https://www.ncbi.nlm.nih.gov/pccompound), PyMol 2.4.0, R (statistical software), R Studio (an integrated development environment for R), STRING (https://stringdb.org/), TCMSP (TCM Systems Pharmacology Database, https://tcmsp-e.com/disease.php?qd=77), TTD (Therapeutic Target Database, https://db.idrblab.net/ttd/), UniProt (http://www.uniprot.org/), Venny 2.1.0 (a tool for comparing different datasets, https://bioinfogp.cnb.csic.es/tools/venny/index.html).

### 2.2. Screening of active ingredients in WMP and target prediction

Active ingredients in WMP were screened using the TCMSP database based on specific criteria 11, including oral bioavailability greater than or equal to 30%, drug-likeness greater than or equal to 0.18, and Caco-2 permeability greater than or equal to −0.4. The identified active ingredients were subsequently linked to their respective target proteins, and further validation and target screening were carried out using the UniProt database.

### 2.3. Selection of UC and T2DM-related targets

Target genes associated with UC and T2DM were gathered from various databases, including CTD, TTD, DrugBank, and GeneCards, through searches for “Ulcerative colitis” and “Type 2 Diabetes.” After removing duplicate and false-positive genes, the common genes shared by both diseases were identified.

### 2.4. Construction of the “Herb-Ingredient-Target-Disease” network and screening of core compounds

To further reveal the complex interactions that underlie the therapeutic effects of WMP in UC and T2DM, the potential target genes were utilized to construct a comprehensive “Herb-Ingredient-Target-Disease” network using Cytoscape 3.9.0. This complex network included nodes representing herbs, herb ingredients, and therapeutic targets, and edges illustrated the multifaceted relationships interconnecting these nodes. In the next phase, this network underwent a rigorous topological analysis facilitated by the Cluego Analyzer software. The analytical approach aimed to unravel the structural intricacies of the network, highlighting key compounds that played pivotal roles in the complex interactions within the network. Core compounds were carefully selected based on their Degree and Betweenness Centrality scores, with a focus on those surpassing the average values.

### 2.5. Construction of protein-protein interaction (PPI) network and selection of core targets

To unravel the intricate mechanisms underlying the therapeutic effects of WMP in UC and T2DM, an in-depth exploration was undertaken using the STRING database. The analysis focused on potential targets, employing criteria such as “Multiple proteins,” with a specific emphasis on the human species. Subsequently, a PPI network diagram was drawn to elucidate the complex interaction network between proteins. In the visualization process, a confidence threshold of 0.4 was set, ensuring the inclusion of only robust interactions. Additionally, proteins lacking substantial connections within the network and those existing in isolation were deliberately excluded to streamline the focus on significant interactions. The resulting PPI network diagram, a comprehensive representation of protein interactions, was then exported for further in-depth analysis of the intricate relationships between target proteins. This subsequent analysis delved into the exploration of PPIs among target proteins, utilizing the sophisticated Cluego Analyzer functionality embedded within Cytoscape 3.9.0 software. This analytical tool facilitated a nuanced network topological analysis, uncovering crucial insights into the pivotal roles played by specific proteins within the network. In the identification of core target points, an emphasis was placed on both Degree and Betweenness Centrality metrics. The top 10 targets in each category were considered as core targets, ensuring a comprehensive understanding of the central nodes in the intricate network of protein interactions.

### 2.6. Enrichment analysis

In order to provide a more comprehensive elucidation of the biological functions and signaling pathways impacted by the intersecting target genes, a detailed examination of the target proteins was conducted through gene ontology (GO) enrichment analysis and Kyoto Encyclopedia of Genes and Genomes (KEGG) enrichment analysis. This process was augmented by a meticulous visualization strategy to enhance clarity. The treatment targets were input into the DAVID database, specifying the species as “homo sapiens” and configuring the list as a gene list. Subsequent to this, a thorough exploration of GO and KEGG enrichment analyses was undertaken to unravel the intricate relationships between the target proteins and their influence on biological processes, cellular components, and molecular functions. In the GO enrichment analysis, a nuanced exploration was conducted, delving into the enriched aspects of biological processes, cellular components, and molecular functions. For the KEGG enrichment analysis, emphasis was placed on selecting results with a significance level of *P* < .01 to ensure robust and meaningful outcomes. After these analyses, in-depth modular analysis of treatment targets was conducted using Metascape and R Studio. This comprehensive analysis sought to extract and delineate potential functional modules associated with the therapeutic effects of WMP in UC and T2DM. The objective was to provide an even more intuitive interpretation of the diverse mechanisms through which each component of WMP may operate within these identified modules.

### 2.7. Molecular docking

To further elucidate the mechanistic underpinnings of WMP in the treatment of UC and T2DM, molecular docking was employed to investigate the interactions between the core compounds of WMP and the central targets implicated in these diseases. The complete protein information was retrieved through the UniProt database, and subsequently, protein structures of core targets with a resolution <2.5 Å, bound to small molecular compounds, were obtained from the PDB database.^[16]^ The 3D structures of the target proteins were downloaded in PDB format. Using PyMol 2.4.0, water molecules and small molecular ligands were removed, resulting in a clean protein structure. Subsequently, AutoDock Tools 1.5.1 software was utilized for hydrogenation and conversion of the protein structure into pdbqt format. Concurrently, the 3D structure of the core component of the WMP was obtained by searching the PubChem database. The SDF format file was imported into OpenBabel to obtain the mol2 format file, which was then imported into AutoDock Tools 1.5.1 software. The ligand underwent charge balancing and hydrogenation processes to generate the pdbqt format file, thereby preparing the ligand for molecular docking with the core target proteins. Grid Box parameters were set for ligand-receptor binding, configuring the system for subsequent molecular docking with the core target proteins. Finally, AutoDock Vina 1.5.1 was employed to simulate molecular docking between the ligand and receptors, and the top 3 results were visualized using PyMol 2.4.0 software.

## 3. Results

### 3.1. Screening and characteristics of active compounds in WMP

In total, 1269 compounds were screened from the TCMSP database using specific criteria, including “OB ≥ 30%, drug-likeness ≥ 0.18, and Caco-2 ≥ −0.4.” Following thorough screening, we identified 90 candidate compounds distributed among the following herbs: 8 from WM, 5 from HJ, 10 from HL, 24 from HB, 5 from GJ, 6 from FZ, 6 from GZ, 8 from XX, 16 from RS, and 2 from DG (Table 1). Collectively, these compounds targeted 1233 effective genes. After standardization and removal of duplicates using the UniProt database, 243 unique gene targets were retained.

**Table 1:** Chemical constituents contained in Chinese herbs in WMP.

Mol ID	Molecule name	OB (%)	DL	Caco-2	Herbal medicine
MOL001040	(R)-naringenin	42.36	0.21	0.38	WM
MOL000358	Beta-sitosterol	36.91	0.75	1.32	WM
MOL000422	Kaempferol	41.88	0.24	0.26	WM
MOL000449	Stigmasterol	43.83	0.76	1.44	WM
MOL005043	Campesterol	37.58	0.71	1.32	WM
MOL008601	Methyl arachidonate	46.9	0.23	1.48	WM
MOL000953	Cholesterol	37.87	0.68	1.43	WM
MOL000098	Quercetin	46.43	0.28	0.05	WM
MOL013271	Kokusaginine	66.68	0.2	0.95	HJ
MOL002663	Skimmianine	40.14	0.2	1.26	HJ
MOL002881	Diosmetin	31.14	0.27	0.46	HJ
MOL000358	Beta-sitosterol	36.91	0.75	1.32	HJ
MOL000098	Quercetin	46.43	0.28	0.05	HJ
MOL001454	Berberine	36.86	0.78	1.24	HL
MOL002894	Berberrubine	35.74	0.73	1.07	HL
MOL002897	Epiberberine	43.09	0.78	1.17	HL
MOL002903	(R)-Canadine	55.37	0.77	1.04	HL
MOL002904	Berlambine	36.68	0.82	0.97	HL
MOL000622	Magnograndiolide	63.71	0.19	0.02	HL
MOL000785	Palmatine	64.6	0.65	1.33	HL
MOL000098	Quercetin	46.43	0.28	0.05	HL
MOL001458	Coptisine	30.67	0.86	1.21	HL
MOL002668	Worenine	45.83	0.87	1.22	HL
MOL001454	Berberine	36.86	0.78	1.24	HB
MOL001458	Coptisine	30.67	0.86	1.21	HB
MOL002643	Delta 7-stigmastenol	37.42	0.75	1.3	HB
MOL002644	Phellopterin	40.19	0.28	0.98	HB
MOL002651	Dehydrotanshinone II A	43.76	0.4	1.02	HB
MOL002662	Rutaecarpine	40.3	0.6	1.13	HB
MOL002663	Skimmianine	40.14	0.2	1.26	HB
MOL002666	Chelerythrine	34.18	0.78	1.24	HB
MOL000449	Stigmasterol	43.83	0.76	1.44	HB
MOL002668	Worenine	45.83	0.87	1.22	HB
MOL002670	Cavidine	35.64	0.81	1.08	HB
MOL002672	Hericenone H	39	0.63	0.8	HB
MOL000358	Beta-sitosterol	36.91	0.75	1.32	HB
MOL000622	Magnograndiolide	63.71	0.19	0.02	HB
MOL000785	Palmatine	64.6	0.65	1.33	HB
MOL000787	Fumarine	59.26	0.83	0.56	HB
MOL000790	Isocorypalmine	35.77	0.59	0.85	HB
MOL000098	Quercetin	46.43	0.28	0.05	HB
MOL001131	Phellamurin	56.6	0.39	0.14	HB
MOL001455	(S)-Canadine	53.83	0.77	1.01	HB
MOL001771	Poriferast-5-en-3beta-ol	36.91	0.75	1.45	HB
MOL002894	Berberrubine	35.74	0.73	1.07	HB
MOL005438	Campesterol	37.58	0.71	1.34	HB
MOL006422	Thalifendine	44.41	0.73	1.12	HB
MOL002464	1-Monolinolein	37.18	0.3	0.32	GJ
MOL002501	(1R,3R)-3-[(E)-3-Methoxy-2-methyl-3-oxo-1-propenyl]-2,2-dimethylcyclopropanecarboxylic acid (S)-3-(2-butenyl)-2-methyl-4-oxo-2-cyclopenten-1-yl	62.52	0.31	0.37	GJ
MOL002514	Sexangularetin	62.86	0.3	0.31	GJ
MOL000358	Beta-sitosterol	36.91	0.75	1.32	GJ
MOL000359	Sitosterol	36.91	0.75	1.32	GJ
MOL002211	11,14-Eicosadienoic acid	39.99	0.2	1.22	FZ
MOL002388	Delphin	57.76	0.28	0.12	FZ
MOL002392	Deltoin	46.69	0.37	0.55	FZ
MOL002395	Deoxyandrographolide	56.3	0.31	0.18	FZ
MOL002398	Karanjin	69.56	0.34	1.22	FZ
MOL000359	Sitosterol	36.91	0.75	1.32	FZ
MOL000358	Beta-sitosterol	36.91	0.75	1.32	DG
MOL000449	Stigmasterol	43.83	0.76	1.44	DG
MOL001736	(-)-Taxifolin	60.51	0.27	-0.24	GZ
MOL000358	Beta-sitosterol	36.91	0.75	1.32	GZ
MOL000359	Sitosterol	36.91	0.75	1.32	GZ
MOL000492	Cianidanol	54.83	0.24	-0.03	GZ
MOL000073	Ent-epicatechin	48.96	0.24	0.02	GZ
MOL004576	Taxifolin	57.84	0.27	-0.23	GZ
MOL012140	4,9-Dimethoxy-1-vinyl-$b-carboline	65.3	0.19	1.21	XX
MOL012141	Caribine	37.06	0.83	0.34	XX
MOL001460	Cryptopin	78.74	0.72	0.79	XX
MOL001558	Sesamin	56.55	0.83	0.75	XX
MOL002501	(1R,3R)-3-[(E)-3-Methoxy-2-methyl-3-oxo-1-propenyl]-2,2-dimethylcyclopropanecarboxylic acid (S)-3-(2-butenyl)-2-methyl-4-oxo-2-cyclopenten-1-yl	62.52	0.31	0.37	XX
MOL002962	(3s)-7-Hydroxy-3-(2,3,4-trimethoxyphenyl)chroman-4one	48.23	0.33	0.62	XX
MOL000422	Kaempferol	41.88	0.24	0.26	XX
MOL009849	Asarinin	31.57	0.83	0.73	XX
MOL002879	Diop	43.59	0.39	0.79	RS
MOL000449	Stigmasterol	43.83	0.76	1.44	RS
MOL000358	Beta-sitosterol	36.91	0.75	1.32	RS
MOL003648	Inermin	65.83	0.54	0.91	RS
MOL000422	Kaempferol	41.88	0.24	0.26	RS
MOL005308	Aposiopolamine	66.65	0.22	0.66	RS
MOL005317	Deoxyharringtonine	39.27	0.81	0.19	RS
MOL005318	Dianthramine	40.45	0.2	-0.23	RS
MOL005320	Arachidonate	45.57	0.2	1.27	RS
MOL005321	Frutinone A	65.9	0.34	0.89	RS
MOL005348	Ginsenoside Rh4	31.11	0.78	0.5	RS
MOL005356	Girinimbin	61.22	0.31	1.72	RS
MOL005376	Panaxadiol	33.09	0.79	0.82	RS
MOL005384	Suchilactone	57.52	0.56	0.82	RS
MOL005399	Sitogluside	36.91	0.75	1.3	RS
MOL000787	Fumarine	59.26	0.83	0.56	RS

DL = drug-likeness, OB = oral bioavailability, WMP = Wumei pills.

### 3.2. Prediction of disease targets and “Herb-Ingredient-Target-Disease” network construction

**Figure 2.**
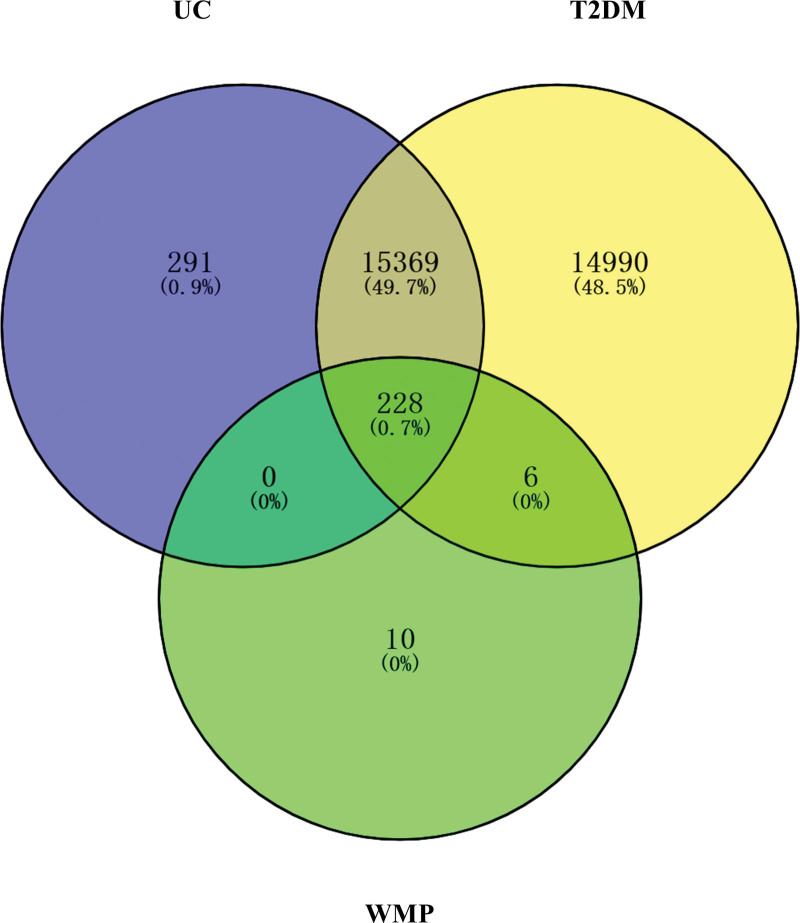
Venn diagram. The blue section represents the targets for UC. The yellow section represents the targets for T2DM. The green section represents the targets for WMP. The common targets among the 3 are 228. T2DM = type 2 diabetes, UC = ulcerative colitis, WMP = Wumei pills.

**Figure 3.**
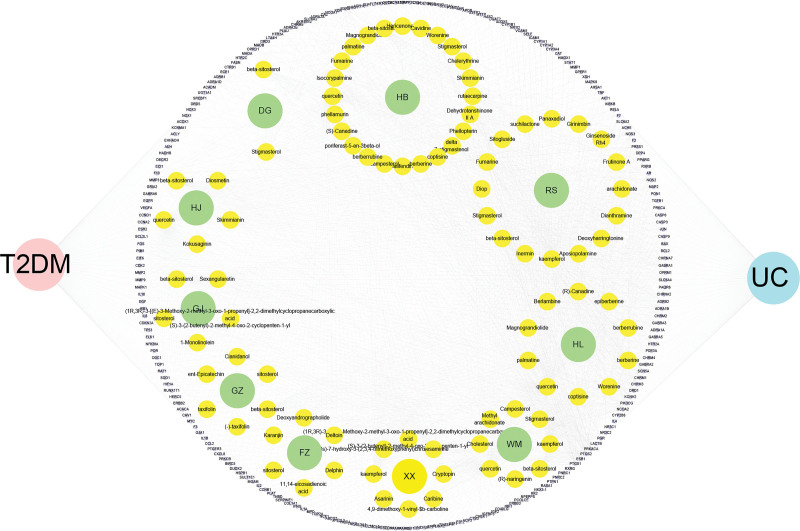
“Herb-Ingredient-Target-Disease” network diagram. Blue circles for UC, pink circles for T2DM, purple circles for targets, green prototypes for herbs, yellow dots for compounds. T2DM = type 2 diabetes, UC = ulcerative colitis.

**Figure 4.**
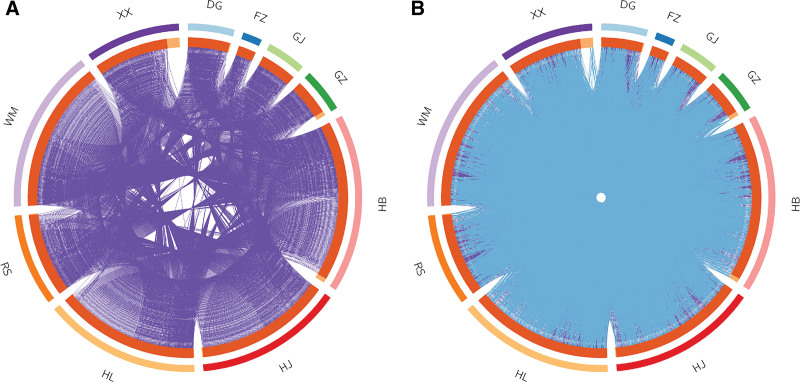
Circos relationship diagram for herbs. The outer arc represented herb ingredients, the inner arc represented genes corresponding to herbs, and the arc length denoted quantity. Various colors were employed to differentiate genes shared among multiple herbs, unique genes exclusive to specific herbs, and purple lines connecting repeated targets (A). Functional repetitions are indicated by blue lines in the right figure (B).

We obtained disease-related targets for UC and T2DM by consulting various disease databases, leading to the identification of 15,888 disease targets for UC and 30,856 for T2DM. The overlapping targets shared between the diseases and WMP treatment consisted of 228 common targets (Fig. 2). We constructed a comprehensive “Herb-Ingredient-Target-Disease” network (Fig. 3). This network was then subjected to Cluego Analyzer analysis. The average Degree was 20.92, and the average Betweenness Centrality was 495.88. Among the compounds, Quercetin, Kaempferol, beta-Sitosterol, Isocorypalmine, and Stigmasterol had values surpassing these averages (Table 2). To visualize the gene overlap in the input lists, a Circos plot was generated using R Studio (Fig. 4).

**Table 2:** Active components with higher than average degree and betweenness centrality.

Herbal compounds	Degree	Betweenness centrality	Herbal medicine
Quercetin	145	6260.35	WM, HJ, HL, HB
Kaempferol	59	1342.71	WM, XX, RS
Beta-sitosterol	37	522.74	WM, HJ, HB, GJ, DG, GZ, RS
Isocorypalmine	33	531.54	HB
Stigmasterol	32	592.43	WM, HB, DG, RS

### 3.3. Selection of core targets

**Figure 5.**
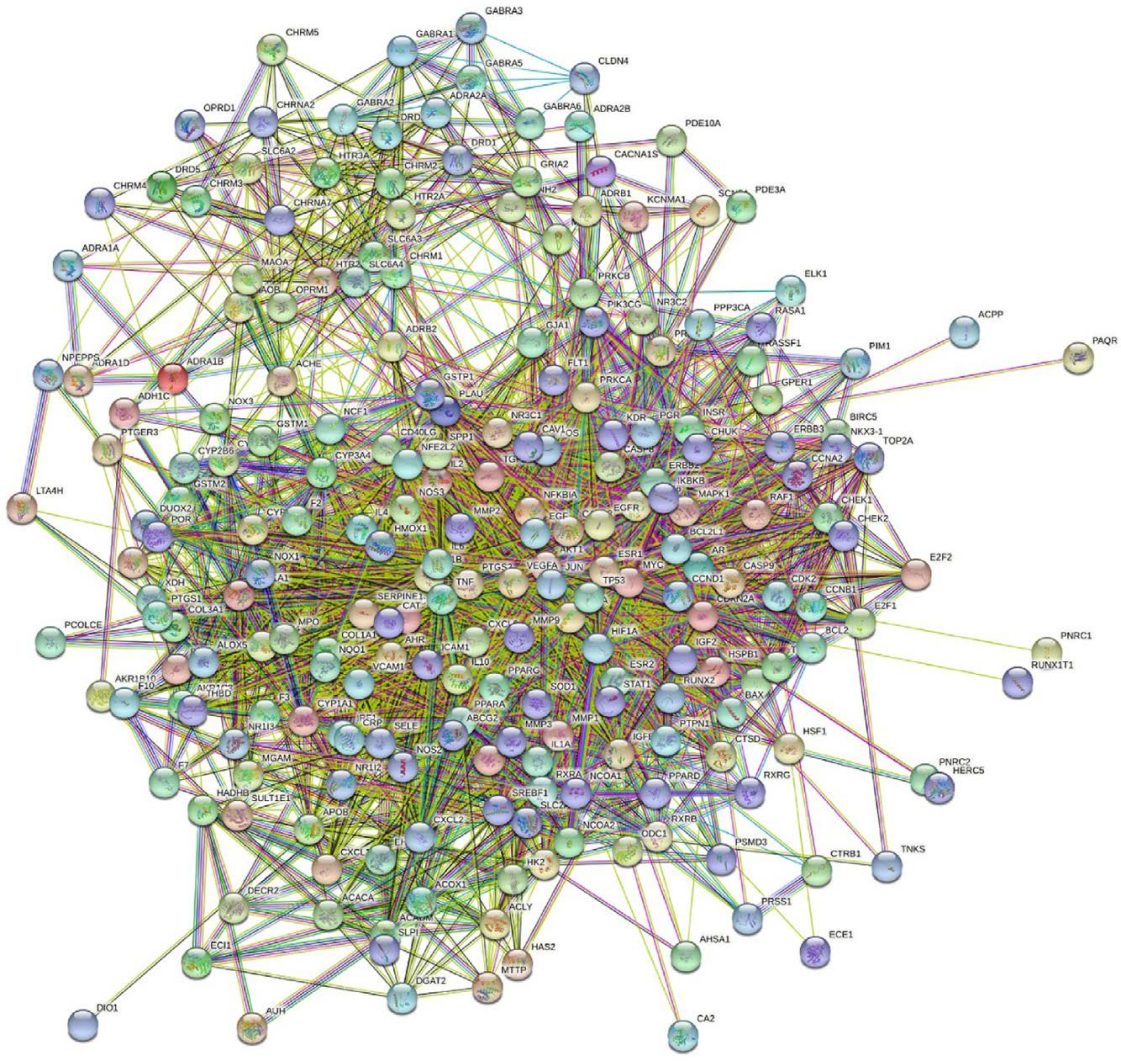
The confidence level of this ppi is 0.4. Freestanding proteins and proteins without mutual connections in the hidden network have been concealed.

**Figure 6.**
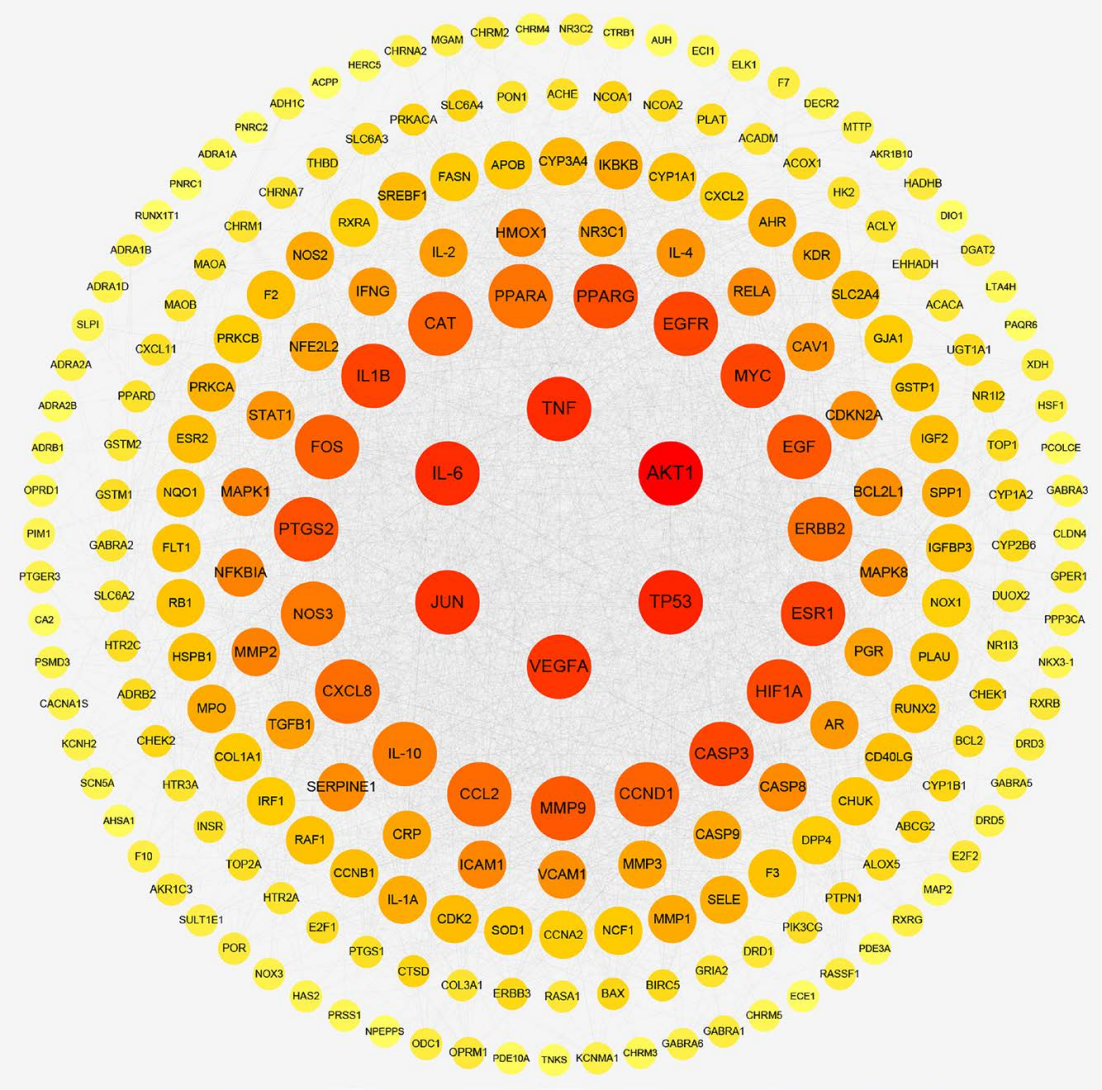
Network diagram of PPI after topological analysis. The higher the number of target interactions, the darker the color.

The 228 common therapeutic targets were used to construct a PPI network within the STRING database, encompassing 227 targets and 3644 edges (Fig. 5). Topological properties of the PPI network were analyzed using the Cluego Analyzer plugin in Cytoscape 3.9.0 (Fig. 6). The top 10 targets, based on both Degree and Betweenness Centrality, were identified as core targets. In total, 8 core targets were selected, including AKT1, ESR1, TP53, IL6, JUN, MYC, TNF, and EGFR (Table 3).

**Table 3:** Information on core target proteins.

Target	Degree	Betweenness centrality
AKT1	130	4057.44
TP53	113	1923.1
IL-6	109	1798.53
TNF	109	1455.9
JUN	106	1641.32
ESR1	99	2483.07
EGFR	97	1324.33
MYC	97	1505.78

### 3.4. Enrichment analysis

To delve deeper into the molecular mechanisms underlying WMP efficacy in treating UC and T2DM, we subjected the target genes to GO biological process analysis and KEGG pathway enrichment analysis using the DAVID and Metascape platforms. Additionally, we employed the MCODE algorithm for modular and enrichment analysis to uncover the functional modules and elucidate herb-specific mechanisms of action.

#### 3.4.1. GO enrichment analysis.

**Figure 7.**
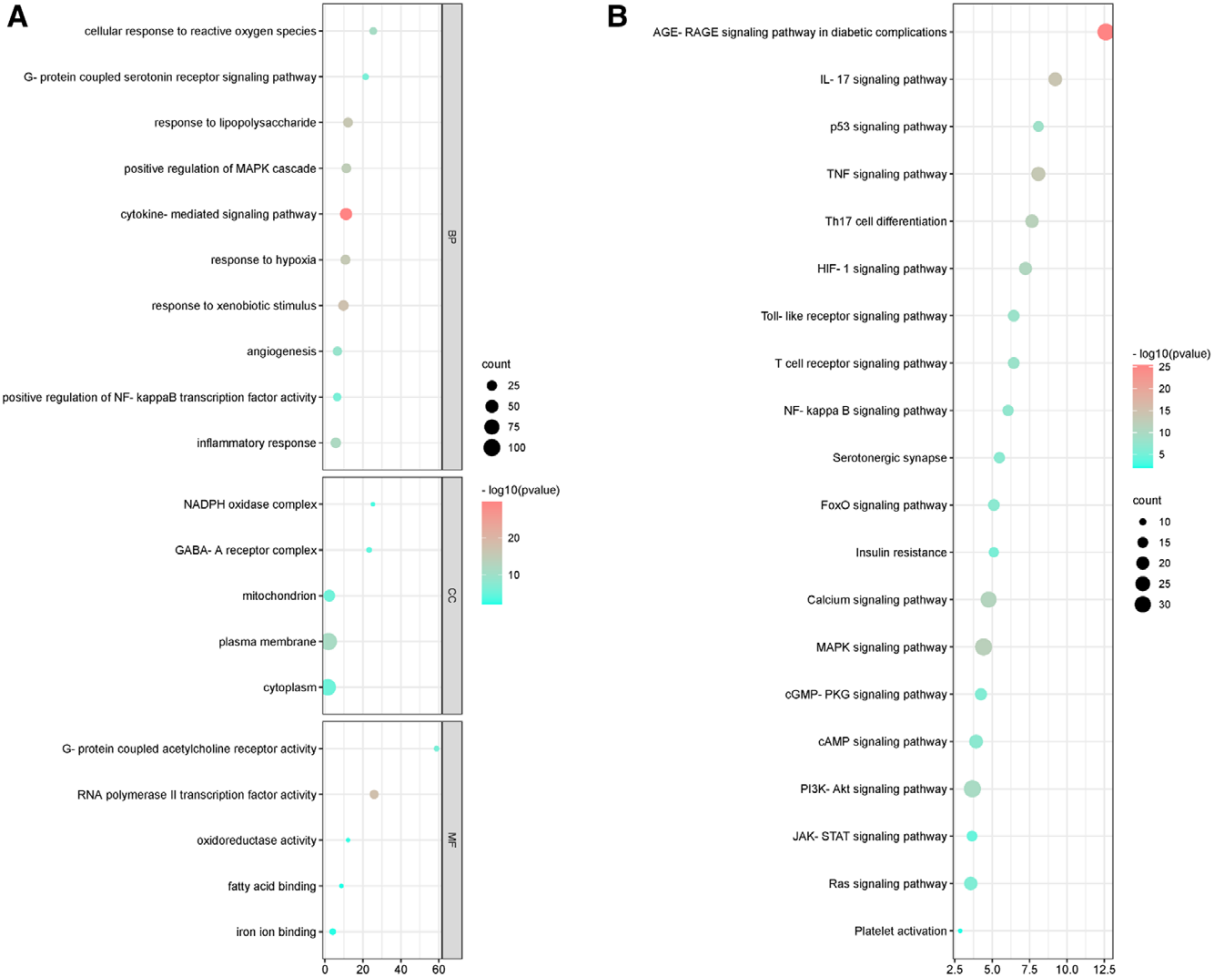
Enrichment analysis bubble diagram. GO enrichment analysis (A). KEGG enrichment analysis (B). GO = gene ontology, KEGG = Kyoto Encyclopedia of Genes and Genomes.

We conducted GO enrichment analysis for the therapeutic targets, yielding the top 20 entries that characterize WMP treatment of UC and T2DM. Notable entries included pathways related to cytokine-mediated signaling, response to lipopolysaccharides, inflammatory responses, and positive regulation of the MAPK cascade (Fig. 7A).

#### 3.4.2. KEGG enrichment analysis.

For a comprehensive understanding of the target genes associated with WMP treatment of UC and T2DM, KEGG enrichment analysis was conducted. The top 20 results with a *P* value below .01 were visualized in a bubble chart (Fig. 7B). The findings indicated that the treatment targets were primarily enriched in pathways such as the “AGE-RAGE signaling pathway in diabetic complications,” “IL-17 signaling pathway,” “p53 signaling pathway,” and “TNF signaling pathway.” These pathways exert substantial influence on the release of inflammatory factors, thereby impacting UC and T2DM.

#### 3.4.3. Modular analysis.

**Figure 8.**
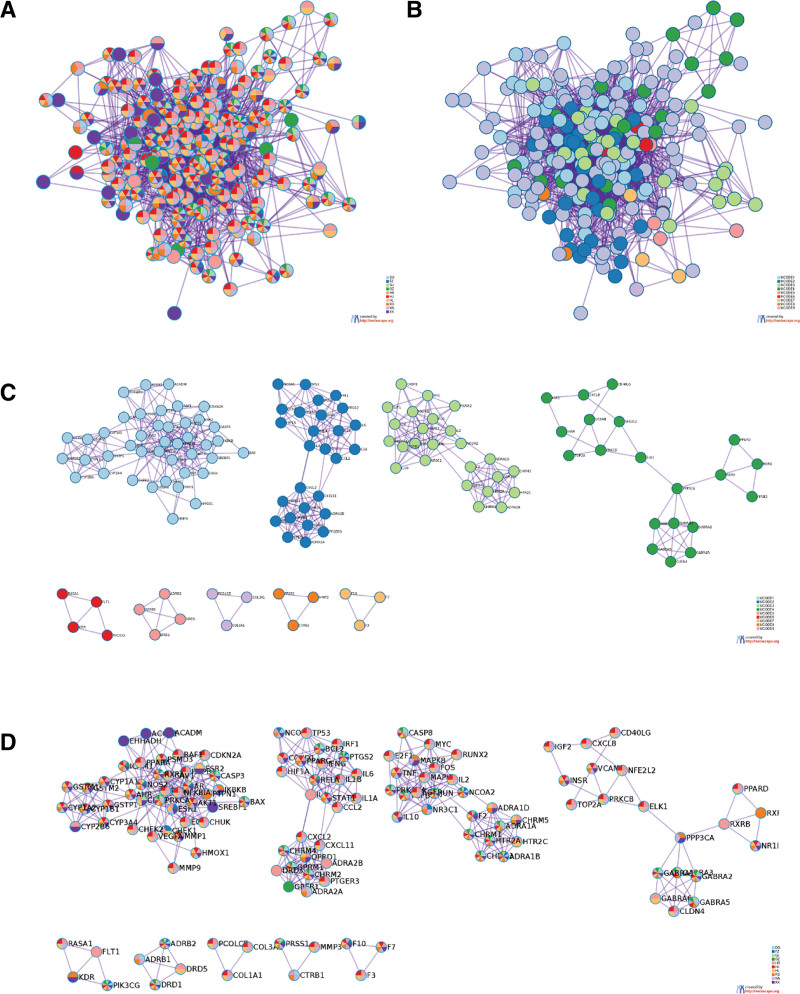
Modular analysis of biological functions.

Cluster analysis results were imported into R Studio for a detailed modular analysis of biological processes. This analysis unveiled the specific actions and significance of each herb within these processes (Fig. 8). We performed GO enrichment analysis for each module network, and the top 3 relevant entries for each MCODE module were analyzed. Our findings indicated that WMP primarily influenced “cellular response to organic cyclic compounds” to intervene in the development of UC and T2DM. The most critical pathway identified was “Pathways in cancer.” When merging the primary biological functions and signaling pathways of each module, the results were consistent with the MCODEALL enrichment analysis, affirming the accuracy of the analysis. Concerning the analysis of individual herbs, DG, GJ, and GZ predominantly influenced the “Neuroactive ligand-receptor interaction” pathway; HB, HJ, HL, and WM mainly acted on “Pathways in cancer”; RS and XX primarily affected “Chemical carcinogenesis—receptor activation”; and FZ primarily targeted “SUMO E3 ligases SUMOylate target proteins.”

### 3.5. Molecular docking results

**Figure 9.**
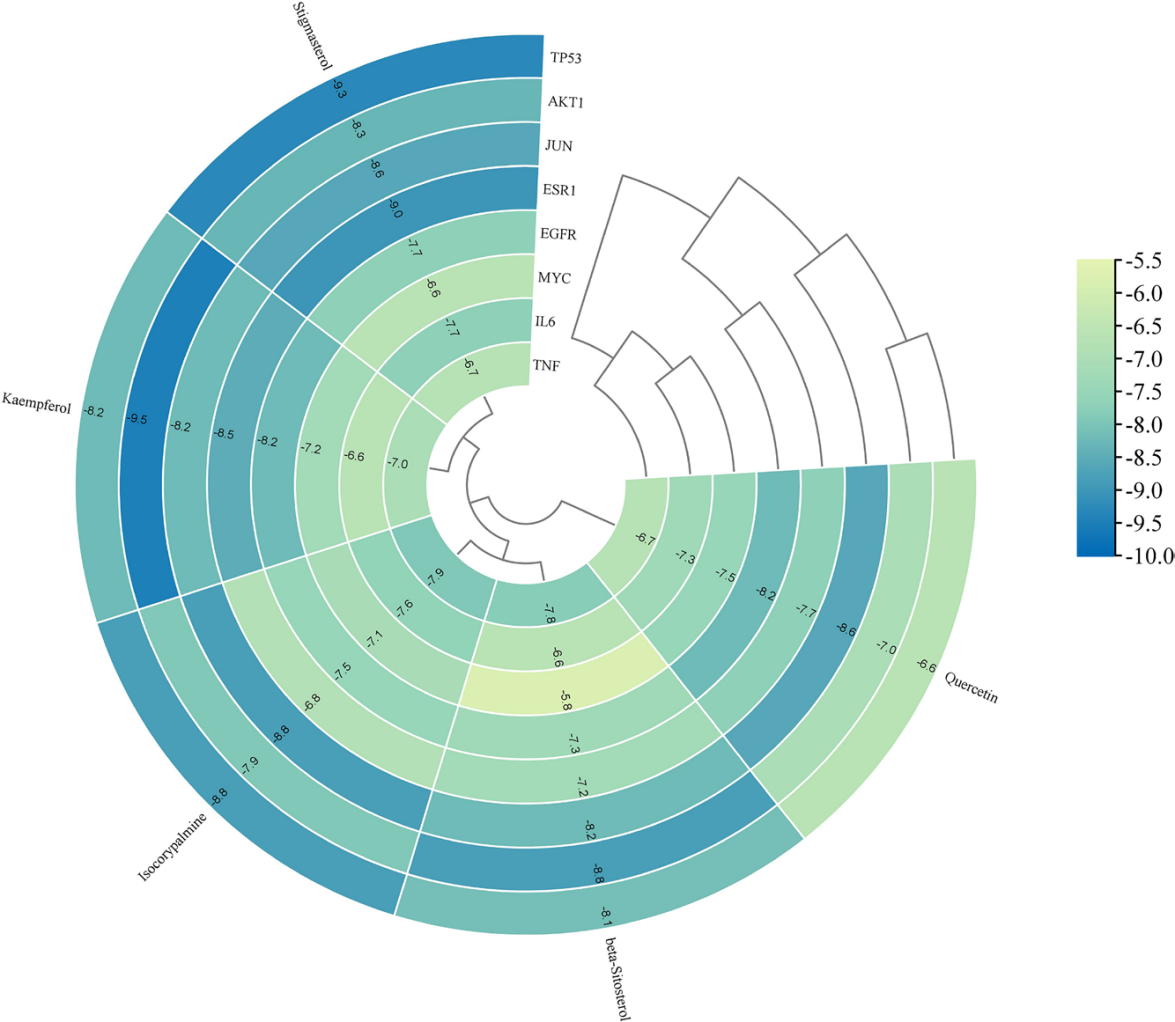
Heat map of molecular docking results. The shift from yellow toward blue indicates an increasingly stable binding between the ligand and the receptor.

**Figure 10.**
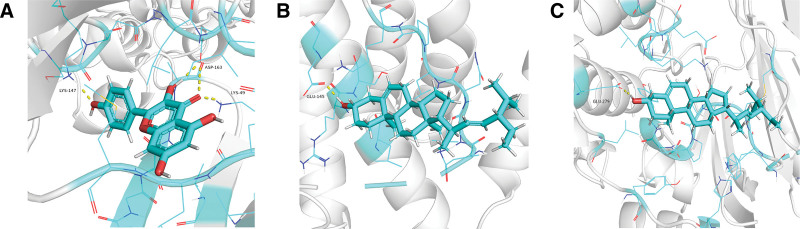
Molecular docking visualization. Kaempferol forms 1 hydrogen bond with AKT1 at LYS-147 and LYS-49, respectively. In addition, they form 2 hydrogen bonds at ASP-163 (A). Stigmasterol forms 1 hydrogen bond with TP53 at GLU-279 (B). Stigmasterol forms 1 hydrogen bond with ESR1 at GLU-145 (C).

The molecular docking technique was employed by us to delve deeper into the intricate interactions between the core compounds of WMP and the central targets implicated in UC and T2DM. The outcomes of this analysis were not merely presented in isolation but were strategically amalgamated and visually represented through the generation of a heat map (Fig. 9). Taking the exploration further, the top 3 docking results were extracted and meticulously visualized using PyMol (Fig. 10). Notably, the average docking score across these interactions was recorded at −7.7 kcal/mol, underscoring the robustness of the binding affinities. Delving into the specificity of the results, an impressive 80.0% of the outcomes displayed docking scores equal to or below −7.0 kcal/mol, affirming the consistently strong binding capabilities observed across the majority of the interactions. Remarkably, the highest stability in docking results was noted in the case of Kaempferol with AKT1, boasting a remarkable score of −9.5 kcal/mol. This exemplifies the exceptional binding affinity between Kaempferol and the AKT1 target, further substantiating the therapeutic potential of WMP in the context of UC and T2DM. In summary, these comprehensive findings underscore the favorable and specific binding capabilities of WMP compounds with their designated targets.

## 4. Discussion

UC, as defined in TCM, falls within the category of conditions such as “chronic diarrhea” and “dysentery.” According to Zhang Jingyue, a prominent TCM scholar, it is believed that “all cases of diarrhea are ultimately related to the spleen and stomach.” Impairment of the spleen functions in transforming and transporting nutrients can result in the accumulation of dampness, eventually leading to the generation of heat. Over time, this may manifest as conditions characterized by diarrhea, urgency, frequent bowel movements, and the presence of mucus or blood in the stool.^[17]^ Similarly, T2DM in TCM falls under categories such as “spleen dampness” and “polydipsia” (excessive thirst). In the “Su Wen: Qi Bing Lun” (Plain Questions: Discussions on Miscellaneous Diseases), it is noted that “Spleen dampness… excess of fatty foods creates internal heat, while sweetness creates a feeling of fullness, and its energy spills upward, causing thirst.” According to TCM principles, overconsumption of fatty and sweet foods can lead to spleen weakness, disruption of energy flow, and irregular distribution of bodily fluids. This can ultimately result in the accumulation of dampness and conditions like polydipsia.^[18]^

Both UC and T2DM are closely intertwined with the concept of the spleen in TCM and are significantly influenced by factors such as “heat” and “dampness.” These shared characteristics provide a strong rationale for adopting a combined treatment approach.^[19]^ In the formulation of WMP, sour-tasting herbs, such as WM, play a vital role in astringing and stopping diarrhea by regulating liver function. Herbs like RS tonify qi and supplement the spleen, while DG nourishes the blood and supplements the liver. Other herbs, such as HL and HB, work together to clear heat, dry dampness, and alleviate heat-related symptoms. When combined, XX, GJ, FZ, HJ, and GZ serve to warm the spleen, invigorate the core, and reinforce kidney yang. They also help balance out the cold properties of herbs like HL and HB. The harmonious combination of these herbs aims to regulate the middle energizer, relieve liver stress, clear heat, dry dampness, and boost qi and blood. WMP is widely used in clinical practice, and numerous researchers have explored its mechanisms.^[14,20–23]^ However, there is currently a gap in research concerning the common mechanisms of action of WMP in treating both UC and T2DM.

WMP consists of 65 different compounds. Among these, Quercetin, Kaempferol, beta-Sitosterol, Isocorypalmine, and Stigmasterol play significant roles in the treatment of UC and T2DM. Quercetin and Kaempferol belong to the flavonoid class of herbs, renowned for their anticancer, anti-inflammatory, antioxidant, and antidiabetic properties.^[24–26]^ For instance, Quercetin can enhance insulin metabolism by activating insulin receptor substrates, thus initiating the PI3K/AKT pathway for improved insulin effects and activating glycogen synthase.^[27]^ Additionally, Quercetin can reduce the levels of pro-inflammatory cytokines like TNF-α, interleukins, and other inflammatory markers to exert its anti-inflammatory effects.^[28]^ Similarly, Kaempferol can impact oxidative stress levels and offer protection to pancreatic islet cells, reducing pancreatic β-cell apoptosis and enhancing insulin secretion while significantly lowering inflammation levels.^[29–32]^ Beta-Sitosterol, on the other hand, regulates insulin resistance and glucose metabolism through various signaling pathways, such as IKKβ, NF-κB, and JNK.^[33]^ Furthermore, it can influence ERK and MAPK signaling pathways to reduce inflammation and lower mRNA expression levels of inflammatory factors like TNF-α and IL-6, thereby improving colon mucosal congestion and edema.^[34,35]^

Core targets, including AKT1, TP53, IL6, and TNF, play pivotal roles within the PPI network. AKT1, as one of the primary subtypes of AKT, acts as a central node in the regulation of cell survival pathways.^[36]^ In the context of UC and T2DM, AKT1 participates in the PI3K/AKT pathway, facilitating metabolic processes, increasing glucose uptake in extracellular tissues by activating downstream molecules such as glucose transporters, and ameliorating insulin resistance.^[37–39]^ Beyond this, AKT1 expression inhibits the polarization of macrophages toward the M1 phenotype, providing anti-inflammatory effects to the gastrointestinal tract.^[40]^ TP53, a downstream protein of P53, becomes engaged in the induction of glycolysis and apoptosis-regulating factors when the P53 pathway is triggered. This protein plays a critical role in oxidative stress responses across various cell types and controls gene expression to regulate inflammatory responses, apoptosis, and genome stability through signaling pathways like PI3K/AKT and NF-κB.^[41]^ The role of IL6 and TNF-α is closely associated with inflammatory responses, participating in the development of inflammation and instigating cellular apoptosis. IL-6 is known to stimulate hepatic glycogenolysis, leading to elevated blood glucose levels.^[42]^ The AGE-RAGE signaling pathway in diabetic complications involves the binding of AGEs to the RAGE, resulting in upregulated MAPK phosphorylation and promoting the expression of inflammatory factors such as TNF-α and IL-6. This pathway triggers oxidative stress and initiates inflammatory reactions.^[43,44]^ Furthermore, lipopolysaccharides can intensify pro-inflammatory factors such as TNF-α and IL-6 by activating signaling pathways like NF-κB and TLR4, thereby exacerbating inflammatory responses.^[45]^

Analysis of the constituent herbs within WMP using Metascape and R Studio software revealed specific pathways influenced by individual herbs. For instance, WM primarily affects signaling by Interleukins and the AGE-RAGE signaling pathway in diabetic complications. Herbs like HJ and XX intervene in pathways related to chemical carcinogenesis—receptor activation, pathways in cancer, interleukin signaling, and cellular response to organic cyclic compounds. On the other hand, HL, HB, FZ, GZ, and GJ play essential roles in neuroactive ligand-receptor interaction, response to hormone, and calcium signaling pathway. Lastly, herbs like DG and RS primarily influence neuroactive ligand-receptor interaction, amine ligand-binding receptors, and G protein-coupled amine receptor activity. The collective actions of WMP and its constituent herbs aim to harmonize the central and liver functions, clear heat, dry dampness, tonify qi, and nourish blood.

## 5. Conclusion and future prospects

In this study, we utilized network pharmacology techniques to analyze and forecast the targets and enriched pathways associated with the effects of WMP in the treatment of UC and T2DM. We identified 5 core compounds, namely Quercetin, Kaempferol, beta-Sitosterol, Isocorypalmine, and Stigmasterol. These compounds were selected for their pivotal roles in the treatment of UC and T2DM. Additionally, we pinpointed 8 core targets: AKT1, ESR1, TP53, IL6, JUN, MYC, TNF, and EGFR. The molecular docking results affirm the presence of strong binding activity between ligands and receptors. This study provides a clear direction and valuable reference for forthcoming research, including mechanistic exploration and experimental validation in the subsequent stages of investigation. This comprehensive analysis sets the stage for further inquiry into the therapeutic potential of WMP in UC and T2DM, offering new insights for the scientific community. We anticipate that the insights derived from this work will contribute to the development of novel treatment strategies for these complex conditions. We look forward to future investigations that will expand upon these findings and lead to a better understanding of the mechanisms underlying WMP efficacy in the treatment of UC and T2DM.

We wish to express our gratitude to all the researchers in the field who have contributed to our collective knowledge. We remain committed to advancing our understanding of these conditions and working toward improved therapies that can enhance the quality of life for affected individuals. This is a promising step toward unlocking the full therapeutic potential of Wumei powder.

## Author contributions

**Writing – original draft:** Chang Sun, Keyuan Xiao, Yinxiong He, Xinghua Li.

**Writing – review & editing:** Yinxiong He.
